# Fatal outcomes among patients on maintenance haemodialysis in sub-Saharan Africa: a 10-year audit from the Douala General Hospital in Cameroon

**DOI:** 10.1186/s12882-016-0377-5

**Published:** 2016-11-03

**Authors:** Marie Patrice Halle, Gloria Ashuntantang, Francois Folefack Kaze, Christian Takongue, Andre-Pascal Kengne

**Affiliations:** 1 Department of clinical sciences, Faculty of medicine and pharmaceutical science, University of Douala, Douala, Cameroon; 2 Department of internal medicine, Douala General Hospital, Douala, Cameroon; 3 Department of internal medicine and specialties, Faculty of medicine and biomedical sciences, University of Yaounde I, Yaounde, Cameroon; 4 South African Medical Research Council and University of Cape Town, Cape Town, South Africa

**Keywords:** ESRD, Outcome, Haemodialysis, Cameroon, Sub-Saharan Africa

## Abstract

**Background:**

End-Stage Renal disease (ESRD) is associated with increased morbidity and mortality. We assessed the occurrence, time-trend and determinants of fatal outcomes of haemodialysis-treated ESRD patients over a 10-year period in a major referral hospital in Cameroon.

**Methods:**

Medical records of ESRD patients who started chronic haemodialysis at the Douala General Hospital between 2002 and 2012 were reviewed. Baseline characteristics and fatal outcomes on dialysis were recorded. Accelerated-failure time and logistic regression models were used to investigate the determinants of death.

**Results:**

A total of 661 patients with 436 (66 %) being men were included in the study. Mean age at dialysis initiation was 46.3 ± 14.7 years. The median [25^th^–75^th^ percentiles] duration on dialysis was 187 [34–754] days. A total of 297 (44.9 %) deaths were recorded during follow-up with statistical difference over the years (*p* < 0.0001 for year by year variation) but not in a linear fashion (*p* = 0.508 for linear trend), similarly in men and women (*p* = 0.212 for gender*year interaction). The death rate at 12 months of follow-up was 26.8 % (*n* = 177), with again similar variations across years (*p* < 0.0001). In all, 34 % of deaths occurred within the first 120 days. Year of study and background nephropathies were the main determinants of mortality, with the combination of diabetes and hypertension conveying a 127 % (95 % CI: 40–267 %) higher risk of mortality, relative to hypertension alone.

**Conclusion:**

Mortality in dialysis is excessively high in this setting. Because most of these premature deaths are potentially preventable, additional efforts are needed to offset the risk and maximise the benefits from the ongoing investments of the government to defray the cost of haemodialysis. Potential actions include sensitisation of the population and healthcare practitioners, early detection and referral of individuals with CKD; and additional subsidies to support the cost of managing co-morbidities in patients with CKD in general.

## Background

Chronic kidney disease (CKD) is a major public health problem and was ranked as the 18^th^ cause of death worldwide in 2010, with most of these deaths occurring in patients with End-Stage Renal Disease (ESRD) [1, 2]. It has been projected that by 2030 more than 70 % of patients with ESRD will originate from low- and middle income countries, such as those in sub-Saharan Africa [3]. ESRD requiring renal replacement therapy (RRT) is the common final pathway for CKD. The population of patients on RRT has doubled in the last two decades. Globally, the number of patients receiving RRT was estimated to be over 1.8 million in 2004, with less than 5 % of this population residing in sub-Saharan Africa (SSA) [4].

Maintenance dialysis therapy is the commonest mode of RRT and demand for this service is increasing with the ESRD population. Although dialysis prevents death from uraemia, patients’ survival remains an important issue. Patients on dialysis have a 3–8 times higher mortality rate than the general population, essentially from increased cardiovascular morbidities [5–7]. There are suggestions that major developments in the management of ESRD patients worldwide have not benefited patients in low- and middle income countries where access to RRT and outcomes of care remain very poor [8–10]. As a result, the mortality rate within 90 days of commencing RRT in SSA countries is as high as 90 %, compared with European countries where it is about 3 % [10, 11]. However existing data from African countries is very patchy and do not reflect the contemporary profile of ESRD patients and patterns of care in this setting.

Dialysis was introduced in Cameroon in the early 1980s and included both peritoneal and haemodialysis, although for over 20 years now haemodialysis has been the only modality of RRT available in the country [12]. For many years however, access to dialysis services in the country has been restricted to few centres (at most three) in the two main cities of the country (Yaoundé and Douala). It is only within the last eight years that the government has expanded haemodialysis services in terms of geographic coverage and capacity to cope with increasing demand. Nevertheless, data regarding the outcome of patients on RRT in Cameroon is inexistent, a situation which is shared by most countries in SSA. The aim of this study was to report on the occurrence, time-trend and determinants of major outcomes of haemodialysis-treated ESRD over a 10 years period in a referral tertiary hospital in Cameroon.

## Methods

### Study setting and clinical pathway of patients with ESRD

This prospective study was carried out in the renal unit of the Douala general hospital (DGH) in Cameroon, and covered the period from 2002 to 2012. DGH is a 320 bedded public institution, which serves as referral hospital for kidney diseases for the Littoral region of the country (approximately 3 million population in 2012) and beyond. The haemodialysis unit of DGH including the staff, equipment and pathway of patients with ERSD, has been described in details previously [13]. At the end of the year 2012, the unit was operating with one nephrologist, two general practitioners and 12 nurses and was equipped with 17 hemodialysis Fresenius® 4008S generators (Fresenius Medical Care, Hamburg, Germany), synthetic polysulfone dialysis membrane, and bicarbonate dialysate was used. Patients in this unit received two weekly sessions of 4 h haemodialysis. Each patient with ESRD has at initiation of dialysis a clinical assessment and laboratory investigations. The register of patients with ESRD starting RRT in the DGH served as basis for patients’ recruitment. This study received administrative authorization from the DGH and was approved by the ethic committee of the Douala University, Cameroon.

The total population in Cameroon was estimated to be about 21.7 million in 2012, with 16.5 % aged less than five years. The total health expenditure was equivalent to 4.3 % of the gross domestic product (GDP), and the general government health expenditures represented about 24.6 % of the total health expenditure and 5.5 % of the general government expenditure [14]. Therefore, in the absence of social security system in Cameroon, out-of-pocket spending represent an important proportion of the health expenditure. However, access to haemodialysis in public health facilities is highly subsidized by the government since 2002, with the co-contribution of patients representing about 5 % of dialysis session cost [13]. In 2012, the country had a total of five nephrologists (two in Douala), eight haemodialysis centres with two in Douala including the study centre and a private haemodialysis centre. The eight centres were providing care to about 500 patients on chronic dialysis, with 30 % (140) of them being managed at the study centre [13].

### Data collection

This study included a total of 661 patients with ESRD on regular haemodialysis at the DGH in Cameroon and was done in the period from the 1^st^ January to 30 June 2013. The case records of all patients with ESRD on RRT over a 10-year period (2002–2012) were retrieved. Data were extracted for each patient on age, sex, major comorbidities (hypertension, diabetes, HIV, gout, history of stroke), the presumed aetiology of ESRD, key parameters at dialysis initiation and the vital status at the last contact with the service.

### Operational definitions

The diagnosis of ESRD was based on the following: estimated glomerular filtration rate using the Cockcroft and Gault formula (from 2002 to 2010) or the four-variable Modification of Diet in Renal Disease (MDRD) formula (from 2010 and beyond) [15, 16] (eGFR) less than 15 ml/min/1.73 m^2^, bilateral shrunken kidneys on ultrasound and presence CKD risk factors and any clinical or biological signs of uraemia. Hypertension, diabetes and HIV were based on documented history, ongoing drug treatments or a documented systolic blood pressure (BP) >140 mmHg and/or diastolic BP >90 mmHg for hypertension or fasting blood glucose >126 mg/dl, or a positive test for HIV.

The background nephropathy was based on clinical arguments in the absence of renal histology data. Chronic glomerulonephritis was based either on a documented history of glomerular disease or the presence of a glomerular syndrome (proteinuria and/or haematuria, hypertension in the absence of identifiable secondary causes). Background nephropathy was ascribed to HIV in the presence of a glomerular syndrome in an HIV positive patient with hyperechogenic and normal size kidneys on ultrasound. Diabetic nephropathy was defined by the presence of hypertension in a diabetic patient associated with glomerular proteinuria, diabetic retinopathy and normal size kidney on ultrasound in the absence of any other cause. CKD was attributed to hypertension in patients with longstanding history of hypertension, and normal urine sediment in the presence of other target organ damages such as left heart hypertrophy, hypertensive retinopathy, and bilateral shrunken kidneys. The aetiology was mixed (diabetes and hypertension) in case of longstanding history of diabetes and hypertension in a patient with clinical sign of both nephropathy. The Charlson comorbidity score was estimated by adding up the scores assigned to existing comorbidities based on the risk of death associated with each of them. The following comorbidities were considered: congestive heart failure, coronary disease, diabetes mellitus, malignancies, stroke, peripheral vascular disease, chronic obstructive pulmonary disease, HIV/AIDS, chronic liver disease and connective tissue disease [17].

Unplanned dialysis was defined as dialysis initiation without preparation, indicated for a life threatening situations using a temporary vascular access (non-tunnelled polyureththane double lumen central venous catheter). Patient’s outcome in this study refers to their status at the last dialysis session – dead or alive. Deaths in the first 120 days of initiation of dialysis were considered as early mortality.

### Statistical analysis

Data were analyzed with the use of SAS/STAT® v 9.1 for Windows (SAS Institute Inc., Cary, NC, USA). We have presented the results as count and percentages, mean and standard deviation (SD) or median and 25^th^–75^th^ percentiles. Differences between men and women and across years of study were investigated via chi square tests and equivalents for qualitative variables, and linearity in the trend across years assessed with the Cochran-Armitage trend test, while the analysis of the variance (ANOVA) and equivalents were used for quantitative variables. The Kaplan-Meier estimator and accelerated failure time models, implemented with the use of LIFETEST and LIFEREG procedures of SAS were used to investigate the baseline characteristics associated with mortality during follow-up. Basic regression models were adjusted for age, sex and year of study, while the extended multivariable models also comprised significant predictors in basic models, based on a threshold for significance of *p* ≤ 0.10. To test the robustness of our findings, determinants were also investigated via logistic regression models, both accounting for the entire duration of follow-up and after restriction of the outcomes to those recorded within the first 12 months of starting the dialysis. To maximize the stability of the estimates across years of study, participants were grouped by clusters of two consecutive years of observations. A *p*-value <0.05 was used to characterize statistically significant results.

## Results

### Sex and age distribution on dialysis overall and by year of study

A total of 661 patients, with 436 (66 %) being men, started RRT during the study period. The proportion of men ranged from 69.2 % in 2002–2004 to 63.6 % in 2011–2012, with a nadir at 61.5 % in 2009–2010, and no significant difference (*p* = 0.575), nor a linear trend (*p* = 0.187 for linearity) across years of study. The mean age was 46.3 (SD = 14.7) years overall and ranged from 46.9 (15.7) years in 2002–2004 to 46.1 (14.5) years in 2011–2012 again with no significant difference, nor a linear trend across years of the study (both *p* ≥ 0.133), and similarly among men and women (*p* = 0.840 for gender*year interaction).

### Profile for chronic kidney disease overall and across years of study

The distribution of the background nephropathy is described in Table 1. Major identified etiologies were hypertension (28.3 %), chronic glomerulonephritis (17.5 %), diabetes mellitus (13.9 %), hypertension and diabetes (7.3 %), HIV (6.7 %), other aetiologies (9.4 %); while no aetiology was identified in 16.9 % of the participants. The distribution of the background nephropathy was similar across years (*p* = 0.622) and in a non-differential way between men and women (*p* = 0.328 for gender*year interaction). The baseline biological profile of patients is also shown in Table 1 indicating the expected high serum urea and creatinine, low hemoglobin and calcium levels, which with the exception of serum urea did not vary across years of study overall (all *p* ≥ 0.240) and by gender (all *p* ≥ 0.649 for gender*year interaction) and did not display a linear trend (all *p* ≥ 0.133 for linearity). Serum urea varied significantly across years (*p* = 0.009) and in a linear fashion (*p* = 0.003 for linearity) from 2.28 g/l in 2002–2004 to 2.80 g/l in 2011–2012. The Median Charlson score was generally around one point score, with suggestion however of significant variations across years (*p* = 0.044) in a linear fashion (*p* = 0.007), but not in a differential way among men and women (*p* = 0.751 for gender*year interaction).

**Table 1:** Profile of patients on dialysis and case-fatality overall and across years of the study

Variables	Overall	2002–2004	2005–06	2007–08	2009–10	2011–12	*p*-value^1^	p-trend^2^	p year*gender^3^
N (%)	661 (100)	120 (18.1)	153 (23.1)	111 (16.8)	156 (23.6)	121 (18.3)			
Men, n (%)	436 (66.0)	83 (69.2)	103 (67.3)	77 (69.4)	96 (61.5)	77 (63.6)	0.575	0.187	NA
Mean age, years (SD)	46.3 (14.7)	46.9 (15.7)	48.3 (13.5)	44.9 (16.4)	45.2 (14.0)	46.1 (14.5)	0.282	0.133	0.840
Mean haemoglobin, g/dl (SD)	7.7 (2.0)	7.4 (2.3)	7.6 (1.9)	7.9 (1.8)	7.5 (1.9)	7.9 (1.9)	0.240	0.521	0.649
Mean Urea, g/l (SD)	2.60 (1.16)	2.28 (0.96)	2.48 (1.17)	2.67 (1.20)	2.74 (1.91)	2.8 (1.1)	0.009	0.003	0.661
Mean creatinine, mg/l (SD)	184.3 (88.9)	168.5 (87.5)	191.7 (104.8)	188.9 (87.3)	184.2 (82.2)	185.1 (75.1)	0.428	0.267	0.702
Mean corrected calcium, mg/l (SD)	80.2 (13.7)	79.6 (14.0)	80.9 (13.7)	82.0 (11.4)	80.9 (13.2)	77.6 (15.6)	0.411	0.523	0.829
Mean phosphate, mg/l (SD)	72.6 (35.2)	85.4 (55.2)	66.0 (30.1)	75.6 (28.0)	66.4 (31.1)	78.2 (33.7)	0.020	0.037	0.270
Planned dialysis start, n (%)	74 (11.2)	8 (6.7)	16 (10.5)	7 (6.3)	23 (14.8)	20 (16.5)	0.031	0.007	0.518
Median dialysis duration, days (Q1-Q3)	187 [37–754]	15 [6–298]	197 [44–836]	218 [57–855]	580 [119–977]	179 [62–357]	<0.0001	0.630	0.173
Background nephropathy, n (%)							0.622	NA	0.328
Hypertension	187 (28.3)	35 (29.2)	40 (26.1)	37 (33.3)	40 (25.6)	35 (28.9)			
Diabetes	92 (13.9)	16 (13.3)	19 (12.4)	18 (16.2)	21 (13.5)	18 (14.9)			
Hypertension and diabetes	48 (7.3)	10 (8.3)	11 (12.4)	8 (7.2)	10 (6.4)	9 (7.4)			
Chronic glomerulonephritis	116 (17.5)	13 (10.8	27 (17.6)	25 (22.5)	34 (21.8)	17 (14.0)			
HIV	44 (6.7)	8 (6.7)	11 (7.2)	5 (4.5)	10 (6.4)	10 (8.3)			
Others	62 (9.4)	9 (7.5)	18 (7.2)	8 (7.2)	16 (10.3)	11 (9.1)			
Unknown	112 (16.9)	29 (21.2)	27 (17.6)	10 (9.0)	25 (16.0)	21 (17.4)			
Median Charlson Score (Q1-Q3)	1 [1–2]	1 [1–2]	1 [1–2]	1 [1–1]	1 [1–1]	1 [1–2]	0.044	0.007	0.751
Death overall, n (%)	297 (44.9)	27 (22.5)	98 (64.0)	55 (49.5)	68 (43.6)	49 (40.5)	<0.0001	0.508	0.212
Death at 12 month, n (%)	177 (26.8)	9 (7.5)	57 (37.2)	33 (29.7)	34 (21.8)	44 (36.4)	<0.0001	0.004	0.138
Death by follow-up duration							<0.0001	NA	0.475
< 120 days	101 (15.3)	8 (6.7)	32 (20.9)	20 (18.0)	20 (12.8)	21 (17.4)			
120–365	76 (11.5)	1 (0.8)	25 (16.4)	13 (11.7)	14 (9.0)	23 (19.0)			
> 365	120 (18.1)	18 (15.0)	41 (26.8)	22 (19.8)	34 (21.8)	5 (4.1)			

*NA* not applicable, Q1–Q3, 25^th^–75^th^ percentiles, *SD* standard deviation

^1^ p-value for year-by-year variation

^2^ p-trend, p-value for the linear trend in the changes across years

^3^ p year*gender, p-value for the interaction effect of gender and year of enrolment on the level of attributes of interest

### Dialysis start and follow-up

In all, the start of dialysis was planned in 74 (11.2 %) participants. This proportion varied from 6.7 % in 2002–2004 to 16.5 % in 2011–2012 (*p* = 0.031). The pattern across years was linear (*p* = 0.007) with no evidence of heterogeneity between men and women (*p* = 0.518 for gender*year interaction). The median duration [25^th^-75^th^ percentiles] on dialysis was 187 [34–754] days overall and varied from 15 [6–298] days in 2002–2004 to 179 [62–357] days in 2011–2012, with a pic of 580 [119–977] in 2009–2010. Variations across years were statistically significant (*p* < 0.0001) but not in a linear fashion (*p* = 0.630), nor in differential ways between men and women (*p* = 0.173 for gender*year interaction), Table 1.

### Mortality and determinants during follow-up

**Fig. 1:**
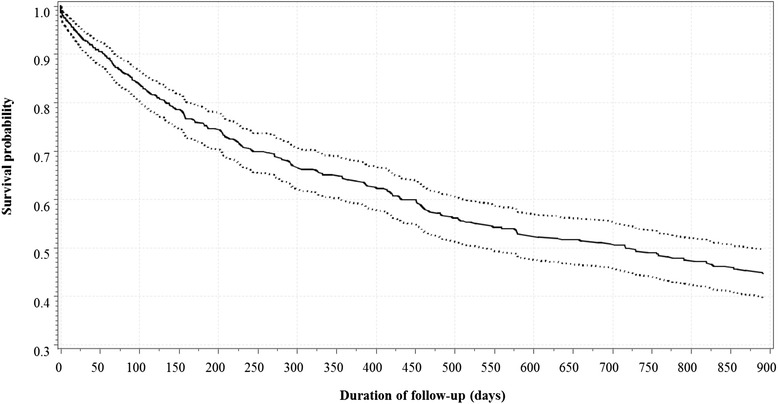
Survival probability from Kaplan-Meir estimator showing the cumulative distribution function (*solid curve*) and the 95 % confidence interval (*dotted lines about*) for mortality during follow-up of patients with end-stage renal disease (ESRD) who started dialysis at the Douala General Hospital between 2002 and 2012. Broken horizontal and vertical lines have been added at regular intervals to assist interpretation

A total of 297 (44.9 %) deaths were recorded during follow-up. This proportion rose from 22.5 % in 2002–2004 to 64.0 % in 2005–2006 and steadily decreased thereafter down to 40.5 % in 2011–2012 (*p* < 0.0001 for year by year variation, and *p* = 0.508 for linear trend), similarly in men and women (*p* = 0.212 for gender*year interaction). The death rate at 12 months of follow-up was 26.8 % (*n* = 177), with again similar variations across years (*p* < 0.0001). Overall, 101 (34 %) deaths occurred within 120 days of starting dialysis; 76 (25.6 %) within 120–365 days and 120 (40.4 %) beyond 365 days, Table 1. The survival probability from the Kaplan-Meier estimator for all-cause mortality is illustrated in Fig. 1.

In sex and age adjusted Weibull regression analyses, year of study (*p* < 0.0001) and background nephropathy (*p* = 0.0001) were significant predictors of mortality while baseline Charlson score (*p* = 0.118) was borderline (Table 2). In further multivariable models adjusted for these factors, year of study and background nephropathy (both *p* < 0.0001) remained the main predictors of mortality during follow-up. Compared with the year 2002–2004, all other years of study were associated with higher risk of mortality with magnitude ranging from 73 to 229 % (Table 2). Using hypertension alone as a referent background nephropathy, diabetes alone was associated with 83 % (26–166 %) higher risk mortality while diabetes combined with hypertension was associated with 127 % (40–267 %) higher risk of mortality. Furthermore ESRD of unknown etiology was associated with 99 % (36–191 %) higher risk of mortality, while with reference to a Charlson score of 1 point, a score of 4 was associated with 111 % (10–307 %) higher risk. The latter however was based on small numbers as indicated by the wide confidence intervals. The pattern was mostly similar, but the magnitude of the association differed (being much higher) when the logistic regression models were applied to investigate the predictors of mortality both for the entire duration of follow-up (Table 3) and after restriction to the first 12 months of follow-up (Data not shown). Furthermore, there was a linear trend in the association of mortality risk with increasing Charlson score (all *p* < 0.07 for linear trend).

**Table 2:** Age sex and year of study adjusted predictors of death from Weibull regression models

Variable	Basic models		Multivariable models	
	HR (95 % CI)	*P*-value	HR (95 % CI)	*P*-value
Age, per year	0.99 (0.99–1.00)	0.412	0.99 (0.98–1.00)	0.517
Sex (men)	0.90 (0.71–1.15)	0.246	0.92 (0.72–1.18)	0.182
Year		<0.0001		<0.0001
2002–2004	1 (reference)		1 (reference)	
2005–2006	2.55 (1.62–3.99)		2.95 (1.85–4.67)	
2007–2008	2.02 (1.24–3.27)		2.40 (1.44–3.98)	
2009–2010	1.49 (0.93–2.39)		1.73 (1.06–2.82)	
2011–2012	2.82 (1.70–4.67)		3.29 (1.94–5.58)	
Background nephropathy		0.0001		<0.0001
Hypertension	1 (reference)		1 (reference)	
Diabetes	1.80 (1.24–2.62)		1.83 (1.26–2.66)	
Hypertension and diabetes	2.31 (1.44–3.72)		2.27 (1.40–3.67)	
Chronic glomerulonephrities	1.08 (0.72–1.62)		1.08 (0.72–1.64)	
HIV	1.63 (0.99–2.69)		1.17 (0.64–2.16)	
Others	1.03 (0.66–1.61)		0.99 (0.63–1.56)	
Unknown	1.97 (1.35–2.87)		1.99 (1.36–2.91)	
Charlson score		0.118		0.124
1	1 (reference)		1 (reference)	
2	1.09 (0.73–1.63)		1.02 (0.67–1.54)	
3	1.23 (0.83–1.82)		1.39 (0.85–2.26)	
4	2.07 (1.10–3.88)		2.11 (1.10–4.07)	
Planned dialysis start	0.86 (0.60–1.23)	0.422	-	
Haemoglobin, per g/dl	0.99 (0.92–1.06)	0.702	-	
Serum creatinine, per 10 mg/l	1.00 (0.98–1.02)	0.884	-	
Calcium, per 10 mg/l	0.96 (0.85–1.08)	0.532	-	
Phosphate, per 10 mg/l	1.01 (0.96–1.07)	0.633	-	

95 % CI, 95 % confidence interval, *HR* hazard ratio

**Table 3:** Age sex and year of study adjusted predictors of death from logistic regression models

Variable	Basic models		Multivariable models	
	OR (95 % CI)	*p*-value	OR (95 % CI)	*p*-value
Age, per year	1.00 (0.99–1.01)	0.844	0.99 (0.97–1.01)	0.282
Sex (men)	0.90 (0.64–1.26)	0.530	0.86 (0.61–1.22)	0.400
Year		<0.0001		<0.0001
2002–2004	1 (reference)		1 (reference)	
2005–2006	6.14 (3.57–10.56)		7.25 (4.13–12.75)	
2007–2008	3.38 (1.91–5.96)		3.86 (2.13–7.00)	
2009–2010	2.64 (1.55–4.49)		2.91 (1.67–5.08)	
2011–2012	2.33 (1.22–4.09)		2.72 (1.52–4.88)	
P for linear trend	0.548		0.451	
Background nephropathy		0.108		0.056
Hypertension	1 (reference)		1 (reference)	
Diabetes	1.94 (1.14–3.30)		2.00 (1.17–3.43)	
Hypertension and diabetes	2.00 (1.02–3.92)		1.99 (1.00–3.95)	
Chronic glomerulonephrities	1.10 (0.63–1.92)		1.08 (0.61–1.92)	
HIV	1.64 (0.81–3.32)		0.81 (0.31–2.14)	
Others	1.19 (0.64–2.18)		1.03 (0.54–1.95)	
Unknown	1.67 (1.00–2.80)		1.76 (1.04–2.99)	
Charlson Score		0.071		0.045
1	1 (reference)		1 (reference)	
2	1.15 (0.66–1.98)		1.12 (0.63–1.98)	
3	1.58 (0.89–2.79)		2.13 (0.96–4.75)	
4	4.03 (1.18–13.79)		5.15 (1.39–18.99)	
P for linear trend	0.013		0.013	
Planned dialysis start	1.27 (0.76–2.11)	0.355		
Haemoglobin, per g/dl	1.00 (0.91–1.10)	0.950	-	
Creatinine, per 10 mg/l	1.00 (0.98–1.02)	0.959	-	
Calcium, per 10 mg/l	1.03 (0.87–1.22)	0.711	-	
Phosphate, per 10 mg/l	1.01 (0.94–1.08)	0.804	-	

95 % CI, 95 % confidence interval, *OR* odd ratio

## Discussion

In this study based on a large sample of patients started on renal replacement therapy for ESRD, we have for the first time in Cameroon and to some extent in SSA, carefully characterised the mortality pattern and determinants over time, using data from a referral dialysis centre. Death rate among patients starting RRT in this setting is unacceptably high, with over a quarter of patients dying within the first year of starting dialysis, and half of those deaths occurring within the first six months. The baseline background nephropathy appears to be a major determinant of mortality, with the combination of diabetes and hypertension conveying an excess risk. Our year-by-year analyses further suggested an improvement over time in the proportion of patients starting RRT following a planned program, but the overall proportion of patients in the category remained unacceptably low.

Mortality in patients commencing or receiving dialysis has been reported in few previous studies in SSA. In a study of 40 incident dialysis cases in Ghana, 32 % of patients died within 90 days of starting dialysis [18]. In a similar study in 91 incident dialysis in Ethiopia, the death rate was 23 % at 90 days, and only 42 % of patients were still alive after 1 year [19]. In a series of 760 ESRD patients managed over a 19-year period (including 565 via dialysis) at a referral hospital in the South-East of Nigeria, 87 % of patients died within the first month of treatment, and at 3 months only 6.8 % of patients receiving haemodialysis were still alive [11]. The 1-year mortality rate was 7.4 % in a cohort of 242 prevalent haemodialysis cases in Sudan [20]. Late presentation (or referral) with CKD and affordability are often cited as major drivers of the high early mortality among patients starting chronic dialysis in SSA.

Differences in dialysis-related medical care influence early dialysis mortality. Interventions identified as important include timely access to pre-ESRD nephrology care, ESRD education and support services, treatment of biochemical abnormalities in advanced CKD, and timely placement of a permanent vascular access. The high early dialysis mortality rate in our study is likely attributable in part to poor quality of care prior to referral, financial constraint, the lack of a national screening and management programs for CKD, the limited number of nephrologists in the country, the high number of late referral [21] and the lack of preparation for dialysis as well as exceptionally high number of unplanned start of dialysis and twice weekly dialysis schedule could explain this situation in our setting [22, 23]. Some other causes of poor outcomes of patients on haemodialysis in Cameroon may include low socioeconomic status of haemodialysis patients, low social support, non-adherence to diet and treatments. Since the year 2002, dialysis is highly subsidized in public health facilities. While, these subsidies cover nearly 95 % of cost of dialysis session, patients and family still have to pay for the costs of all concurrent treatments, laboratory investigations and incident hospitalisations; which for the majority of patients, are unaffordable [13]. Studies elsewhere have highlighted catheter use and late or absent pre-ESRD nephrology care, as well as malnutrition as captured by hypoalbuminemia, as major ‘reversible’ determinants of early dialysis mortality [24, 25].

Although some existing studies from Africa have collected data across years, none has presented sufficiently time-trend data for comparisons. The largest study is from Arogundade et al. in Nigeria [11], and has covered a 19-year’s period (1989–2007). In this study, the background nephropathy appeared to be similar across time periods; in line with our findings of no significant variation in background nephropathies across years. We however observed significant variations in mortality rates across years, not explained by varying lengths of exposure to dialysis, and other study characteristics. The apparent low mortality rate in the first years of study likely reflect incomplete follow-up. This is reflected by the very short duration of follow-up on dialysis, implying that patients were transferred to other centres soon after the start of the dialysis, dropped out of dialysis, or perhaps died in other settings. Between 2005 and 2010, death rate mostly declined, likely reflecting the better staffing of the service with initially one nephrologist, then subsequently two. Further increase in mortality rate in the last years of study are likely explained by severe shortage of consumables for dialysis experienced by the service and other centres across the country during those 2 years. Over the years covered by the study, conditions to access dialysis in public institutions have mostly remained the same. However issues with the supply chain for dialysis consumable, which occurred in differential ways across years, could be a major driver of differences in mortality across years. We also noted that an increasing proportion of patients started dialysis in planned manners across years; however their absolute number was very small to affect early mortality [26].

We found background nephropathy to be a determinant of the overall and early mortality, with the combination of diabetes and hypertension conferring very high risk, followed by diabetes alone, unknown aetiologies and glomerulonephritis. In general, people with diabetes tend to have many co-morbidities, are at high risk of fatal infections on temporary vascular access, cardiovascular and all-cause mortality. In general, the poor survival in dialysis of patients with diabetes is well documented [27, 28]. Patients with ESRD of unknown aetiology are also more likely to carry a high burden of co-morbidities, some of which on their own (like undiagnosed cancers) are highly fatal regardless of RRT. Across existing studies from Africa, advanced uraemia, cardiovascular diseases and infections have variably been reported as causes of death in patients starting dialysis [11, 19, 22, 23].

### Strengths and limitations

Our study has some limitations. Analyses are based on data found in records, therefore precluding the investigation of the broad spectrum of predictors of mortality in dialysis, as well as the whereabouts of the defaulters. Furthermore, we completely lacked data on causes of death which were not assessed in this study. Data were collected from a single centre, which could raise issues regarding the generalizability to the entire country. However, the study centre is the only public institution to provide sufficient data for the range of time covered in the current study. The background nephropathy in these patients mostly reporting with advanced CKD was essentially based on clinical arguments. Such an approach is not all accurate considering for instance that the differentiation between hypertension causing ESRD and hypertension resulting from or co-occurring with ESRD may not be possible from a cross-sectional analysis. Our study also has major strengths including the large sample size and the long period of data collection. Our study is one of the largest to provide time-trend on the outcomes of contemporary patients commencing hemodialysis in SSA, and using robust analytic methods.

## Conclusion

In conclusion, mortality in patients with ESRD commencing dialysis in our setting is very high, with about 15 % of patients dying within the first 120 days of starting haemodialysis. In spite of some improvement over times, the overall and early mortality rates have remained unacceptably high in these patients, and far above rates reported in developed countries. Because most of these premature deaths are potentially preventable, additional efforts are needed in this setting to offset the risk and maximise the benefits from the ongoing investments of the government to defray the cost of haemodialysis. Potential actions include intensive sensitisation of the population and healthcare practitioners, in order to improve early detection and referral of individuals with CKD; and additional subsidies to support the cost of managing co-morbidities in patients with CKD in general.

## Abbreviations

AIDS: Acquired immunodeficiency syndrome
ANOVA: Analysis of the variance
CKD: Chronic kidney disease
DGH: Douala General Hospital
ERSD: End-stage renal disease
HIV: Human immunodeficiency virus
MDRD: Modification of Diet in Renal Disease
RRT: Renal replacement therapy
SD: Standard deviation
SSA: Sub-Saharan Africa.
